# PD-1 expression, among other immune checkpoints, on tumor-infiltrating NK and NKT cells is associated with longer disease-free survival in treatment-naïve CRC patients

**DOI:** 10.1007/s00262-022-03337-8

**Published:** 2022-11-27

**Authors:** Mohammad A. Al-Mterin, Khaled Murshed, Eyad Elkord

**Affiliations:** 1 Natural and Medical Sciences Research Center, University of Nizwa, P.O. Box 33, 616 Nizwa, Oman; 2 Department of Pathology, Hamad Medical Corporation, Doha, Qatar; 3 Department of Biological Sciences and Chemistry, Faculty of Arts and Sciences, University of Nizwa, Birkat Al Mouz, 616 Nizwa, Oman; 4 Biomedical Research Center, School of Science, Engineering and Environment, University of Salford, Manchester, UK

**Keywords:** Colorectal cancer, Natural killer cells, Natural killer T cells, Programmed cell death-1, Disease-free survival

## Abstract

A variety of variables, such as microsatellite instability or inflammatory mediators, are critical players in the development and progression of colorectal cancer (CRC). Natural killer (NK) and natural killer T (NKT) cells are involved in the prognoses of CRC. Immunological components of the tumor microenvironment (TME) impact cancer progression and therapeutic responses. We report that CRC patients with higher frequencies of tumor-infiltrating PD-1^+^ NK and NKT cells had significantly longer disease-free survival (DFS) than patients with lower frequencies. In agreement with that, patients with higher frequencies of tumor-infiltrating PD-1^−^ NK and NKT cells showed shorter DFS. There were no significant associations between tumor-infiltrating PD-1^+^TIM-3^+^, PD-1^+^TIGIT^+^, PD-1^+^ICOS^+^, PD-1^+^LAG-3^+^ NK cells, and PD-1^+^TIM-3^+^, PD-1^+^TIGIT^+^, and PD-1^+^LAG-3^+^ NKT cells with DFS. This study highlights the significance of PD-1 expression on tumor-infiltrating NK and NKT cells and its association with disease prognoses in CRC patients.

**Supplementary Information:**

The online version contains supplementary material available at 10.1007/s00262-022-03337-8.

## Introduction

Colorectal cancer (CRC) is one of the most prevalent causes of cancer-related death [1]. Cytokine pathways are critical components, particularly in the case of colorectal cancer, where inflammation and antitumor immunity are primary factors determining disease progression [2]. In addition, chronic inflammation in colon tissues could increase CRC incidence [3].

Many different types of immune cells make up the human immune system. Natural killer (NK) cells, T lymphocytes, B lymphocytes, and other cells all play important roles in the host immune system’s ability to combat tumors [4]. NK cells are innate immune cells that are characterized by their quick and powerful cytolytic activity in response to infected or mutated cells. There is a diverse collection of receptors on their surface that may have either inhibitory or stimulatory functions [5]. Also, they are responsible for regulating the immune response via the production of cytokines including interferon-alpha (IFN-alpha) and tumor necrosis factor alpha (TNF-alpha) [6]. In CRC, it has been shown that a higher percentage of NK cells in the peripheral blood indicated a longer survival time [7]. Interestingly, a study found that patients with low NK cell activity had a 10-time higher risk of CRC, compared with patients with high NK cell activity [8]. Another study found that NK cell receptor ligands expressed by CRC cells may modify the phenotype of circulating NK and NKT cells, allowing cancer cells to evade the immune system [9].

NKT cells are a distinct subset of T cells, which are located at the boundary between the innate and the adaptive immune systems. NKT cells, as opposed to ordinary T cells, express a T cell receptor (TCRs) that is capable of recognizing glycolipids that are presented via an HLA-like protein [10]. Upon activation, NKT cells secrete large amounts of either pro- or anti-inflammatory cytokines, resulting in amplification or inhibition of the immune responses [11].

We have recently reported that some inhibitory or stimulatory receptors, including programmed cell death-1 (PD-1), T cell immunoreceptor with Ig and ITIM domains (TIGIT), T cell immunoglobulin and mucin domain-containing protein-3 (TIM-3), cytotoxic T lymphocyte-associated antigen (CTLA-4), inducible T cell costimulatory (ICOS), and lymphocyte-activation gene-3 (LAG-3), are expressed on T cells, B cells, and NK cells and play a significant role in CRC disease progression [12, 13]. In this study, we investigated correlations of immune checkpoints (ICs) expression on NK and NKT cells in the TME with disease-free survival (DFS). This is the first study to report potential associations between frequencies of NK and NKT cells expressing ICs with DFS in CRC patients.

## Materials and methods

### Patients and samples

This study was approved by the Institutional Review Board at Hamad Medical Corporation (HMC) (Study no. MRC-02-18-012), Doha, Qatar. All patients provided informed consent before collecting the samples. Tumor and normal tissues were taken from 45 treatment-naïve CRC patients at various cancer stages (stage I–IV). Patients who received neoadjuvant chemotherapy before the surgery were excluded from the study. Clinical and pathological characteristics of these patients are shown in Table 1. Patients with TNM stage I/II did not receive any treatment after the surgery. However, based on the American Cancer Society guidelines, patients with stage III/IV (with lymph node metastasis and/or distant organ metastasis) were given adjuvant chemotherapy following the surgery.

**Table 1:** Clinical and pathological features of the study cohort

	CRC patients
Number	45
Median age [range]	56 [18–79]
Gender [Male/Female]	30:15
*TNM stage*	
I	4
II	20
III	14
IV	7
*Tumor histological grade*	
G2 (Moderately differentiated)	41
G3 (Poorly differentiated)	4
*MSI-H/dMMR*	8
Loss of nuclear expression for MLH1 & PMS2	7
Loss of nuclear expression of MSH2	1
*Molecular testing*	
KRAS mutations	4
BRAF mutations	1
NTRK gene fusion	1

*CRC* Colorectal cancer, *MSI-H/dMMR* high microsatellite instability /deficient mismatch repair

Normal tissues were sampled from the wall of colon resected for the same patient. The section from the normal tissue was taken from a distance more than 10 cm away from the tumor. Cell suspensions from normal and tumor tissues were prepared by mechanical disaggregation using gentleMACS Dissociator (Miltenyi Biotec, Bergisch Gladbach, Germany) without using any enzymes. To remove aggregates and debris, cells were passed through Falcon 100 μM cell strainers. Single-cell suspensions were washed with PBS and stained before flow cytometric analyses.

### Multi-parametric flow cytometry

In this study, no extra experiments were performed, and flow cytometric data of both NK cells and NKT cells were collected. According to the protocol outlined in our previously published work, immune staining and flow cytometry analyses have been performed [13]. Briefly, cells were washed and suspended in flow cytometry staining buffer. FcR Blocker (Miltenyi Biotec) was used to block Fc receptors (FcR) on cells. After that, cells were stained with antibodies against different markers. Viability staining solution (7-Aminoactinomycin D, 7-ADD, BioLegend, San Diego, CA, USA) was used to gate live cells. Cell surface antibodies against CD3-BrilliantTM Ultraviolet 496 (BUV496; clone UCHT-1; BD Biosciences, Oxford, UK), CD56-Phycoerythrin (PE; clone B159; BD Biosciences), CD4-Fluorescein Isothiocyanate (FITC; clone RPA-T4; BD Biosciences), PD-1-PE/DazzleTM 594 (clone EH12.2H7; BioLegend), ICOS-Alexa Fluor 700 (AF700; clone C398.4A; BioLegend), LAG-3-Brilliant violet 421 (clone T47-530; BD Biosciences), TIM-3-Brilliant Violet 711 (BV711; clone 7D3; BD Biosciences), and TIGIT-APC (clone MBSA43; eBioscience) were added, and cells were incubated at 4 °C for 30 min. BD LSRFortessa X-20 SORP flow cytometer was used to analyze the samples. After that, BD FACSDiva software (BD Biosciences) was used to collect the data. Then, the collected data was analyzed by FlowJo V10 software (FlowJo, Ashland, OR, USA). According to the gating of the flow cytometric plots, NK cells were identified as CD3^−^CD56^+^, and NKT cells as CD3^+^CD56^+^, as previously described [13, 14].

### Statistical analyses

GraphPad Prism 9 software (GraphPad Software, California, USA) was utilized to perform the statistical analyses. The normality of datasets was carried out by using Shapiro–Wilk test. All cell subsets were divided into low and high groups as lower/higher than the mean for normally distributed data, and lower/higher than the median for non-normally distributed data. Kaplan–Meier curves were used to estimate the DFS, and *P* values were calculated by using the log-rank test. Paired/unpaired t tests were performed based on distribution of data and the normality of datasets, for comparisons within and between groups, respectively. *P* value of ≤ 0.05 was considered to be statistically significant.

## Results

### Association of immune checkpoint-expressing NK cells with DFS

**Fig. 1:**
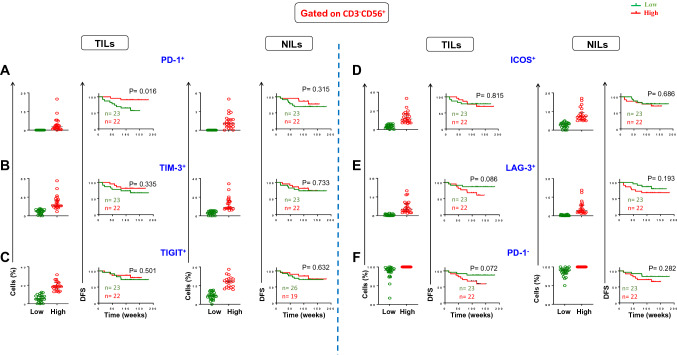
Scatter plots and Kaplan–Meier curves of DFS based on frequencies of different immune checkpoint expression in TILs and NILs. Patients with high frequencies of PD-1^+^ (**A**), TIM-3^+^ (**B**), TIGIT^+^ (**C**), ICOS^+^ (**D**), LAG-3^+^ (**E**), PD-1^−^ (**F**), and LAG-3^−^ (**G**) in CD3^−^CD56^+^ NK cells were compared with those with low frequencies of these cells

Recently, numerous studies have observed that several ICs can be expressed on TILs, as well as they have critical effects on their functions in cancer progression [12, 13, 15]. In this study, we first investigated any potential association between expression of different ICs on NK cells and DFS in TILs and NILs (as controls) in CRC patients. We divided patients into two groups as below and above mean/median levels of these cells (PD-1^+^: TILs ^median^ 0, NILs ^median^ 0; TIM-3^+^: TILs ^median^ 15.5, NILs ^median^ 5.8; TIGIT^+^: TILs ^median^: 12.5, NILs ^mean^ 11.6; ICOS^+^: TILs ^median^ 6.3, NILs ^median^ 5; LAG-3^+^: TILs ^median^ 1.9, NILs ^median^ 1.2). Of note, 23 patients had no expression of PD-1 on NK cells (Fig. 1A, scatter plot). Among the five ICs investigated, we found for the first time that PD-1 expression on NK cells was significantly associated with DFS. CRC patients with higher frequency of PD-1^+^ NK cells in TILs had significantly longer DFS than patients with lower frequency of these immune cells (Fig. 1A). In agreement with that, lack of expression of PD-1 on NK cells in TILs showed a trend toward shorter DFS (Fig. 1F). Additionally, there were no associations between other IC expressions and DFS (Fig. 1B–E).

We then investigated if co-expression of other ICs with PD-1 can be associated with DFS. Interestingly, we did not find any significant association between tumor-infiltrating PD-1^+^TIM-3^+^, PD-1^+^TIGIT^+^, PD-1^+^ICOS^+^, and PD-1^+^LAG-3^+^ NK cells in TILs and NILs in CRC patients (Supplementary Fig. S1). These findings indicate that co-expression of different immune checkpoints did not add any value, and PD-1 expression on NK cells could be an independent prognostic biomarker for DFS in CRC patients.

### Association of immune checkpoint-expressing NKT cells with DFS

**Fig. 2:**
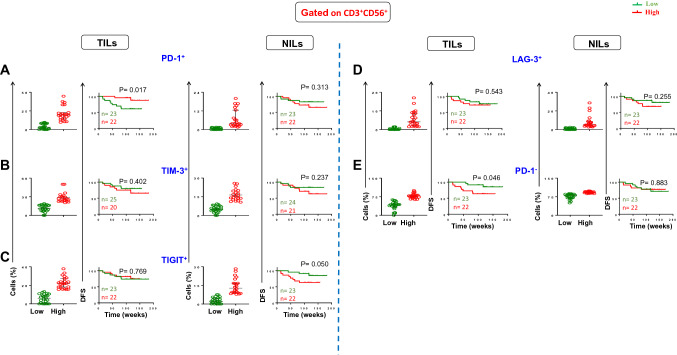
Scatter plots and Kaplan–Meier curves of DFS based on frequencies of different immune checkpoint expression in TILs and NILs. Patients with high frequencies of PD-1^+^ (**A**), TIM-3^+^ (**B**), TIGIT^+^ (**C**), LAG-3^+^ (**D**), PD-1^−^ (**E**), and LAG-3^−^ (**F**) in CD3^+^CD56^+^ NKT cells were compared with those with low frequencies of these cells

We did similar investigations for any possible associations between immune checkpoint-expressing NKT cells and DFS (Fig. 2). We divided patients into two groups as below and above mean/median levels of these cells (PD-1^+^: TILs ^median^ 8.0, NILs ^median^ 1.9; TIM-3^+^: TILs ^mean^ 19.1, NILs ^mean^ 10.3; TIGIT^+^: TILs ^median^ 13.2, NILs ^median^ 7.5; LAG-3^+^: TILs ^median^: 1.46, NILs ^median^: 2.51). Interestingly, we found for the first time that CRC patients with higher frequency of tumor-infiltrating PD-1^+^ NKT cells had significantly longer DFS than patients with lower frequency of these immune cells (Fig. 2A). In agreement with that, lack of expression of PD-1 on NKT cells was significantly associated with shorter DFS (Fig. 2E). There were no significant associations between TIM-3^+^, TIGIT^+^, and LAG-3^+^ expressing NKT cells and DFS (Fig. 2B–D).

We then investigated the effect of co-expression of other ICs with PD-1 on DFS. We divided patients into two groups as below and above mean/median levels of these cells (PD-1^+^TIM-3^+^: TILs ^median^ 15.5, NILs ^median^ 0; PD-1^+^TIGIT^+^: TILs ^median^ 9.5, NILs ^median^ 0.4; PD-1^+^LAG-3^+^: TILs ^median^ 0.5, NILs ^median^ 0). We did not find any significant association between co-expression of PD-1 and other ICs with DFS in TILs and NILs (Supplementary Fig. S2). These findings indicate that co-expression of different immune checkpoints did not have any additional value, and PD-1 expression on NKT cells could be an independent prognostic biomarker for DFS in CRC patients.

### CRC patients with MSI-H have higher levels of tumor-infiltrating PD-1^+^ NK cells

Colorectal cancer patients with deficient mismatch repair (dMMR)/microsatellite instability-high (MSI-H) tumors have better prognosis and overall survival than patients with microsatellite-stable (MSS)/microsatellite instability-low (MSI-L) tumors [16, 17]. This is attributed to considerable differences of infiltrating immune cells between both groups. It has been found that dMMR/MSI-H tumors have microenvironment rich in cytotoxic CD8^+^ T cells (CTLs) and PD-L1^+^ cells [16]. Therefore, MSI-H tumors have shown higher sensitivity to ICIs than MSS/MSI-L tumors [17, 18]. Despite that, it has been found that many patients with dMMR/MSI still respond poorly to immunotherapies. This is likely due to intratumor heterogeneity resulting from complex mechanisms such as frequent immunoediting of WNT/β-catenin signaling, antigen presentation machinery, and IFN-γ signaling [18]. In our study, 8 out of the 45 patients (~ 18%) had dMMR/MSI-H tumors. As expected, we found that CRC patients with MSI-H have longer DFS than patients with MSI-L (data not shown), which is in an agreement with other studies [19–21]. Then, we investigated ICs expression on NK cells in CRC patients based on MSI status in the TME (Supplementary Fig. S3). We found that frequency of PD-1^+^ NK cells in CRC patients with MSI-H was significantly higher, compared to patients with MSI-L (median (95% CI); 2.35 (0–5.35) vs. 0 (0–0.75), *P* = 0.032). This supports our finding that PD-1 expression on NK cells is associated with good prognoses. There were no differences in levels of TIM-3^+^, TIGIT^+^, and LAG-3^+^ NK cells between MSI-H and MSI-L tumors in our cohort. Patients with MSI-H tumors showed lower levels of ICOS^+^ NK cells than patients with MSI-L tumors (Supplementary Fig. S3).

## Discussion

Several inhibitory checkpoints have been identified on T cells and NK cells, such as PD-1, TIGIT, CD96, TIM-3, and others [13, 22, 23]. For example, Yang et al. reported that TIGIT^+^PD-1^+^ T cells are unable to stimulate effective immune responses against tumors [15]. In addition, PD-1^+^TIM-3^+^ TILs show the most highly exhausted phenotype in the TME [24]. Moreover, a study observed that intratumoral NK cells expressed high levels of PD-1 on their cell surface in NSCLC patients. Also, they found that intratumoral NK cells were less functional compared to peripheral NK cells, and this dysfunction correlated with PD-1 expression [25]. These checkpoints are crucial for maintaining the homeostasis of immune cell subsets [22]. Under pathological conditions, some of these checkpoint regulators, which are absent on resting NK cells, may be spontaneously triggered by contact with certain ligands that are widely expressed on the surface of cancer cells, impairing antitumor NK cell function and facilitating tumor immune escape [26]. PD-1 expression was detected on NK cells in peripheral blood of patients in several types of cancer, such as multiple myeloma [27], renal cell carcinoma patients [28], and Hodgkin lymphoma [29]. In addition, tumor-infiltrating PD-1^+^ NK cells were identified in non-small cell lung cancer, where these cells co-expressed more inhibitory receptors, as compared to the PD-1^−^ subset [25]. Liu et al*.* [30] found that elevated expression of PD-1 on NK cells indicated poorer survival in esophageal and liver cancers. However, in inflammatory cancers such as CRC, high secretion of inflammatory cytokines from activated NK cells could be a critical factor to determine disease progression [2, 31].

A study found that after antigenic stimulation, PD-1 expression was increased on invariant NKT cells [32, 33]. In a melanoma model, PD-1 inhibition restored NKT cell proliferation and cytokine production, resulting in potent antitumor responses [32, 34]. Moreover, in non-small cell lung cancer (NSCLC) patients, PD-1 expression was increased on NKT cells, leading to reduced proliferation capacity compared with controls [33]. Additionally, it has been found that PD-1 and LAG-3 were predominantly upregulated on NKT and CD8^+^ T cells in NSCLC patients [35]. Until now, the role of NKT expressing ICs on DFS in CRC patients remains largely unknown.

In our study, high frequencies of tumor-infiltrating PD-1^+^ NK cells and PD-1^+^ NKT cells in the TME were associated with longer DFS, indicating the beneficial anti-inflammatory role of these cells in the TME of CRC patients. Interestingly, we did not find any significant association between co-expression of PD-1 with other ICs on NK and NKT cells with DFS in TILs and NILs, suggesting that the co-expression of different immune checkpoints did not help to further improve the role of other ICs in the prognoses of CRC patients. Importantly, PD-1 expression on NK and NKT cells could be a potential prognostic biomarker for DFS in CRC patients.

To the best of our knowledge, this is the first study to show the significance of PD-1 expression on tumor-infiltrating NK cells and its association with prolonged DFS in CRC patients. Overall, our findings highlight the significance of PD-1 expression on NK and NKT cells, which could be potential predictive biomarkers for CRC disease prognoses and therapeutic approaches. However, there are some limitations in this study. Multicenter investigations are required to confirm these findings in larger cohorts of patients. Additionally, more investigations and multivariable analyses are needed to determine the exact function of these cells in the TME of CRC patients. Findings with *P* values near 0.05 should be interpreted carefully. Additionally, defining NK cells in tissues as CD3^−^CD56^+^ does not discriminate between NK cells and innate lymphoid cells (ILCs) and this cell population could be both NK cells and ILCs [36]. Deep phenotyping of NK cells and NKT cells using different markers such as CD57, CD16, NKG2A, KIRs, and NCRs is essential to identify the exact subsets of these cells in the TME.

### Supplementary Information

Below is the link to the electronic supplementary material.

**Fig. S1**: **Kaplan–Meier curves of DFS based on frequencies of PD-1 co-expression with other immune checkpoints in TILs, and NILs**. Patients with high frequencies of PD-1^+^TIM-3^+^ (**A**), PD-1^+^TIGIT^+^ (**B**), PD-1^+^ICOS^+^ (**C**), and PD-1^+^LAG-3^+^ (**D**) in CD3^−^CD56^+^ NK cells, were compared with those with low frequencies of these cells. Supplementary file1 (PPTX 170 kb)

**Fig. S2**: **Kaplan–Meier curves of DFS based on frequencies of PD-1 co-expression with other immune checkpoints in TILs, and NILs**. Patients with high frequencies of PD-1^+^TIM-3^+^ (**A**), PD-1^+^TIGIT^+^ (**B**), PD-1^+^LAG-3^+^ (**C**) in CD3^+^CD56^+^ NKT cells, were compared with those with low frequencies of these cells. Supplementary file2 (PPTX 134 kb)

**Fig. S3**: **Scatter plots of frequencies of different immune checkpoint expression in MSI-H versus MSI-L tumors.** Scatter plots show PD-1^+^ (**A**), TIM-3^+^ (**B**) TIGIT^+^ (**C**), ICOS^+^ (**D**), and LAG-3^+^ **(E)** in tumor-infiltrating NK cells in MSI-H and MSI-L tumors. Supplementary file3 (PPTX 226 kb)

## Data Availability

The datasets used and/or analyzed during the current study are available from the corresponding author on reasonable request.

## Abbreviations

CRC: Colorectal cancer
CTLA-4: Cytotoxic T lymphocyte-associated antigen 4
DFS: Disease-free survival
ICs: Immune checkpoints
ICOS: Inducible T cell costimulatory
LAG-3: Lymphocyte-activation gene 3
NILs: Normal tissue-infiltrating lymphocytes
NK: Natural killer cells
NKT: Natural killer T cells
PD-1: Programmed cell death-1
TIGIT: T cell immunoreceptor with Ig and ITIM domains
TILs: Tumor-infiltrating lymphocytes
TIM-3: T cell immunoglobulin and mucin domain-3
TME: Tumor microenvironment
