# Supplementary material for: PD-1 expression, among other immune checkpoints, on tumor-infiltrating NK and NKT cells is associated with longer disease-free survival in treatment-naïve CRC patients
Source: Cancer Immunol Immunother. 2022 Nov 27;72(6):1933–9. doi: 10.1007/s00262-022-03337-8 (PMC10198836; doi:10.1007/s00262-022-03337-8)
Supplement: Supplementary file 1 — Fig. S1: Kaplan–Meier curves of DFS based on frequencies of PD-1 co-expression with other immune checkpoints in TILs, and NILs. Patients with high frequencies of PD-1+TIM-3+ (A), PD-1+TIGIT+ (B), PD-1+ICOS+ (C), and PD-1+LAG-3+ (D) in CD3−CD56+ NK cells, were compared with those with low frequencies of these cells. Supplementary file1 (PPTX 170 kb) [file 262_2022_3337_MOESM1_ESM.pptx]

## Slide 1
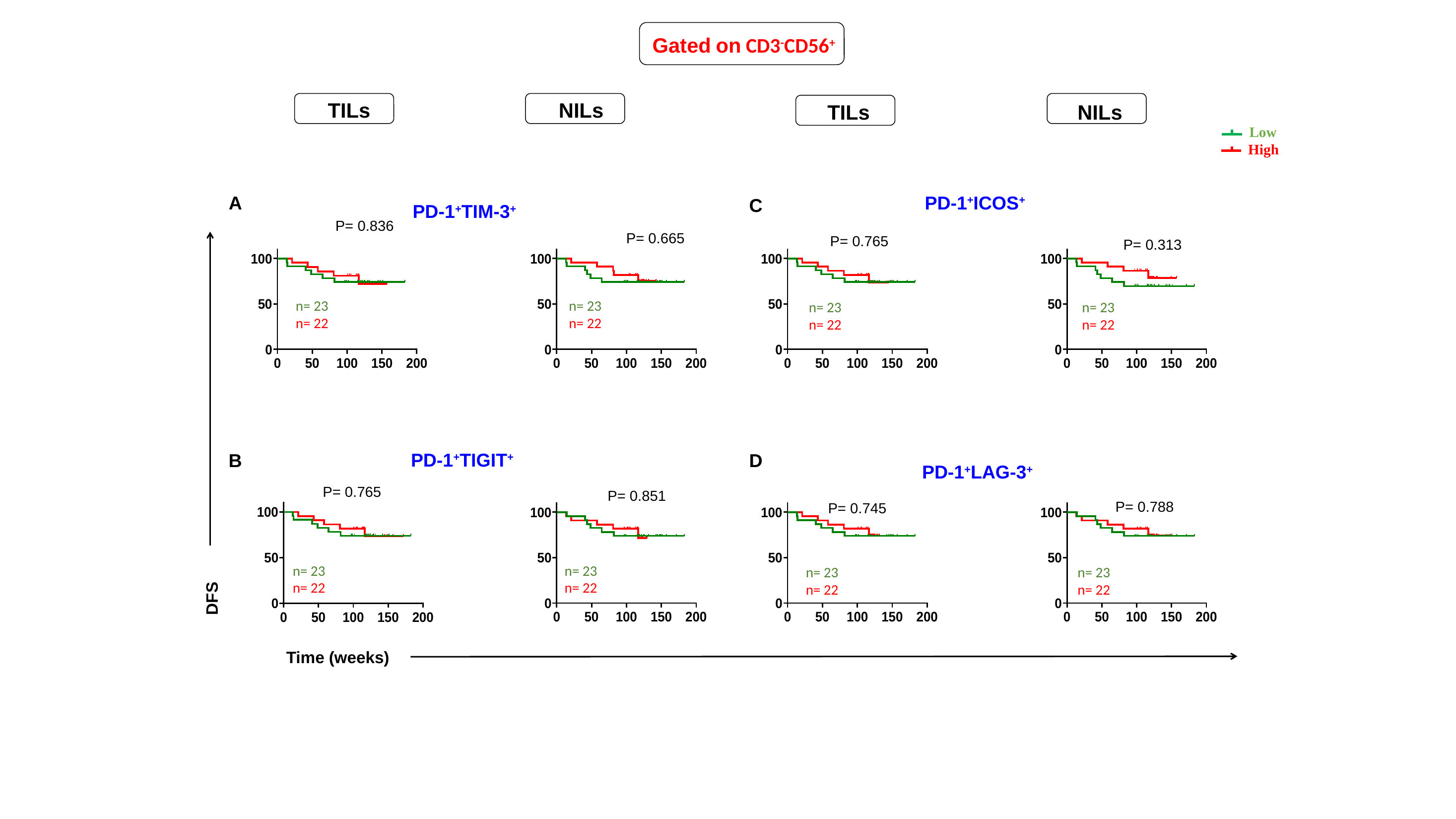

Gated on CD3-CD56+
TILs
NILs
TILs
NILs
 Low
High
PD-1+ICOS+
A
C
PD-1+TIM-3+
P= 0.836
P= 0.665
P= 0.765
P= 0.313
n= 23
n= 22
n= 23
n= 22
n= 23
n= 22
n= 23
n= 22
PD-1+TIGIT+
B
D
PD-1+LAG-3+
P= 0.765
P= 0.851
P= 0.788
P= 0.745
n= 23
n= 22
n= 23
n= 22
n= 23
n= 22
n= 23
n= 22
DFS
Time (weeks)
