# Supplementary material for: PD-1 expression, among other immune checkpoints, on tumor-infiltrating NK and NKT cells is associated with longer disease-free survival in treatment-naïve CRC patients
Source: Cancer Immunol Immunother. 2022 Nov 27;72(6):1933–9. doi: 10.1007/s00262-022-03337-8 (PMC10198836; doi:10.1007/s00262-022-03337-8)
Supplement: Supplementary file 2 — Fig. S2: Kaplan–Meier curves of DFS based on frequencies of PD-1 co-expression with other immune checkpoints in TILs, and NILs. Patients with high frequencies of PD-1+TIM-3+ (A), PD-1+TIGIT+ (B), PD-1+LAG-3+ (C) in CD3+CD56+ NKT cells, were compared with those with low frequencies of these cells. Supplementary file2 (PPTX 134 kb) [file 262_2022_3337_MOESM2_ESM.pptx]

## Slide 1
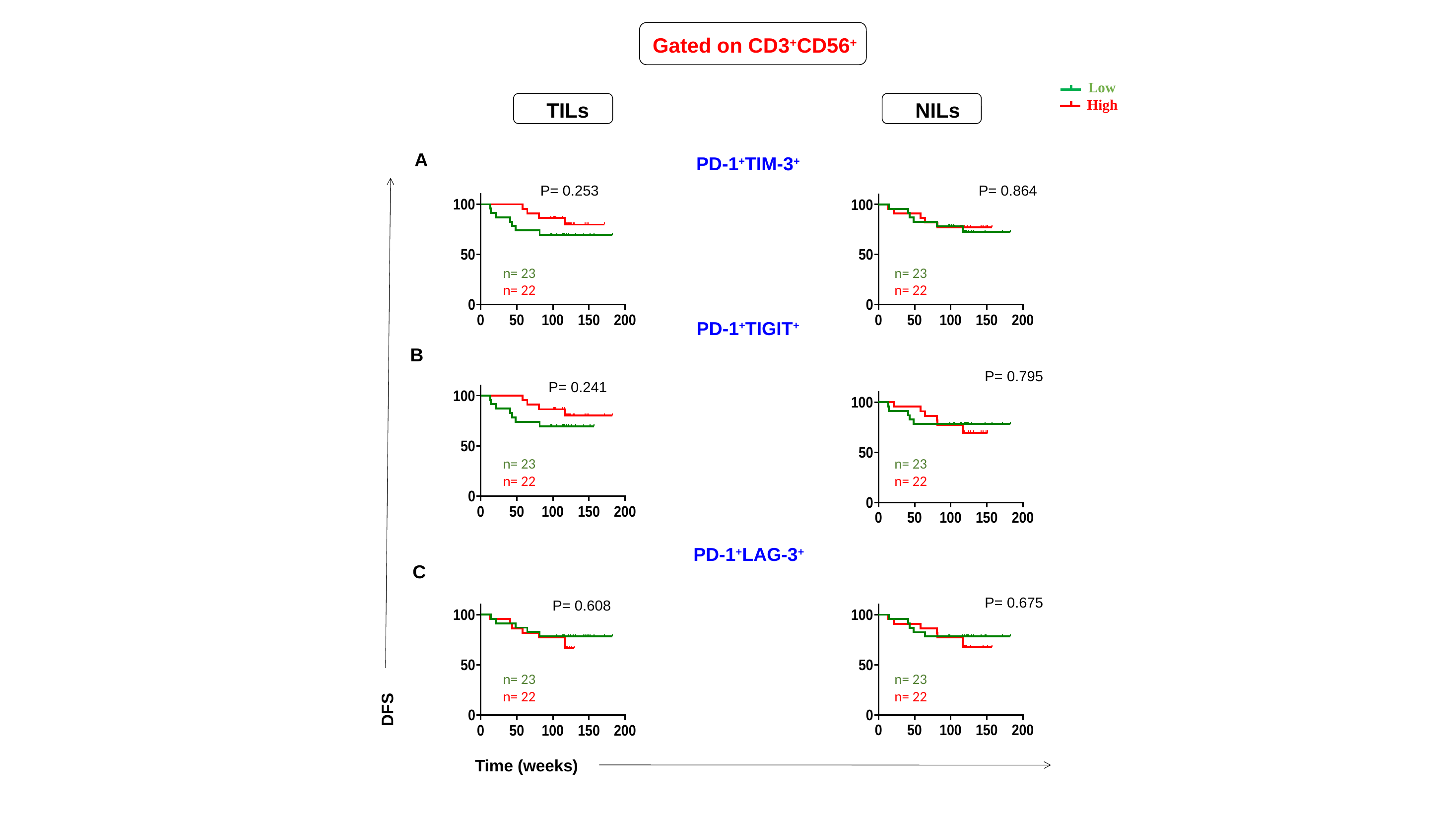

Gated on CD3+CD56+
 Low
High
TILs
NILs
A
PD-1+TIM-3+
P= 0.253
P= 0.864
n= 23
n= 22
n= 23
n= 22
PD-1+TIGIT+
B
P= 0.795
P= 0.241
n= 23
n= 22
n= 23
n= 22
PD-1+LAG-3+
C
P= 0.675
P= 0.608
n= 23
n= 22
n= 23
n= 22
DFS
Time (weeks)
