# Supplementary figures and images for: PD-1 expression, among other immune checkpoints, on tumor-infiltrating NK and NKT cells is associated with longer disease-free survival in treatment-naïve CRC patients
Source: Cancer Immunol Immunother. 2022 Nov 27;72(6):1933–9. doi: 10.1007/s00262-022-03337-8 (PMC10198836; doi:10.1007/s00262-022-03337-8)

## Slide 1
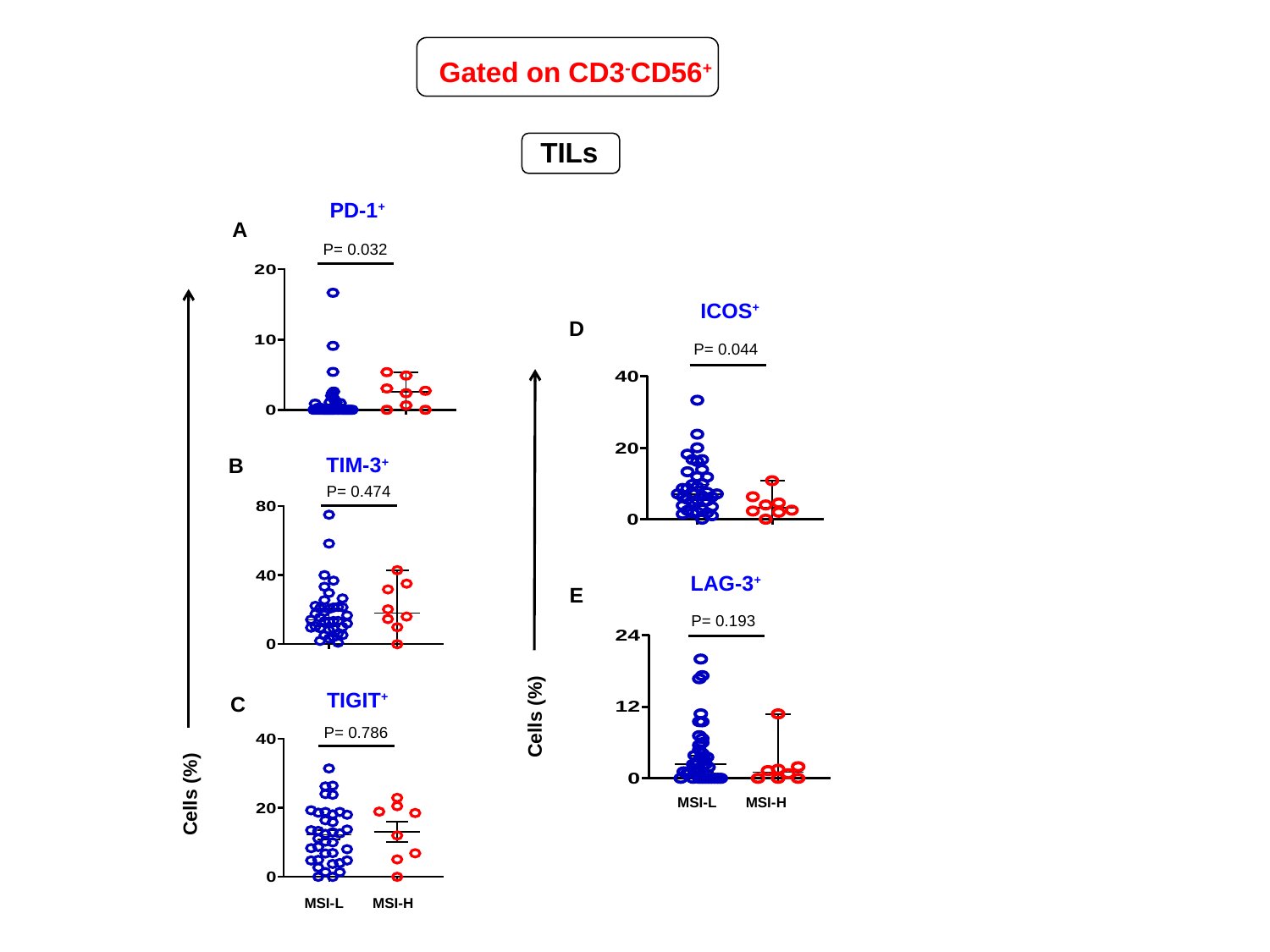

Gated on CD3-CD56+
TILs
PD-1+
A
P= 0.032
ICOS+
D
P= 0.044
TIM-3+
B
P= 0.474
LAG-3+
E
P= 0.193
TIGIT+
C
Cells (%)
P= 0.786
Cells (%)
MSI-L
MSI-H
MSI-L
MSI-H

Supplement: Supplementary file 3 — Fig. S3: Scatter plots of frequencies of different immune checkpoint expression in MSI-H versus MSI-L tumors. Scatter plots show PD-1+ (A), TIM-3+ (B) TIGIT+ (C), ICOS+ (D), and LAG-3+ (E) in tumor-infiltrating NK cells in MSI-H and MSI-L tumors. Supplementary file3 (PPTX 226 kb) [file 262_2022_3337_MOESM3_ESM.pptx]
